# Self-Assembly, Surface Activity and Structure of *n*-Octyl-β-D-thioglucopyranoside in Ethylene Glycol-Water Mixtures

**DOI:** 10.3390/ijms14023228

**Published:** 2013-02-05

**Authors:** Cristóbal Carnero Ruiz, José Antonio Molina-Bolívar, José Manuel Hierrezuelo, Esperanza Liger

**Affiliations:** Department of Applied Physics II, Engineering School, University of Málaga, Málaga 29071, Spain; E-Mails: jmb@uma.es (J.A.M.-B.); jose.hierrezuelo@uma.es (J.M.H.); eliger@uma.es (E.L.)

**Keywords:** *n*-octyl-β-d-thioglucopyranoside, ethylene glycol, surface tension, light scattering, time-resolved fluorescence anisotropy

## Abstract

The effect of the addition of ethylene glycol (EG) on the interfacial adsorption and micellar properties of the alkylglucoside surfactant *n*-octyl-β-d-thioglucopyranoside (OTG) has been investigated. Critical micelle concentrations (cmc) upon EG addition were obtained by both surface tension measurements and the pyrene 1:3 ratio method. A systematic increase in the cmc induced by the presence of the co-solvent was observed. This behavior was attributed to a reduction in the cohesive energy of the mixed solvent with respect to pure water, which favors an increase in the solubility of the surfactant with EG content. Static light scattering measurements revealed a decrease in the mean aggregation number of the OTG micelles with EG addition. Moreover, dynamic light scattering data showed that the effect of the surfactant concentration on micellar size is also controlled by the content of the co-solvent in the system. Finally, the effect of EG addition on the microstructure of OTG micelles was investigated using the hydrophobic probe Coumarin 153 (C153). Time-resolved fluorescence anisotropy decay curves of the probe solubilized in micelles were analyzed using the two-step model. The results indicate a slight reduction of the average reorientation time of the probe molecule with increasing EG in the mixed solvent system, thereby suggesting a lesser compactness induced by the presence of the co-solvent.

## 1. Introduction

The interest in elucidating the role of polar solvents, other than water, in the self-assembly of amphiphiles, and the structure of the aggregates formed in these media, has increased steadily over the last decades. The pioneering work of Evans and co-workers on micelle formation in hydrazine established that the specific characteristics of water are not a requisite for promoting surfactant self-assembly [1]. Indeed, a number of polar organic solvents with strong cohesive forces, such as ethylene glycol, glycerol, or formamide, which have been widely investigated in this context, are also able to induce aggregation [2]. In addition to high cohesive forces, these solvents have a high dielectric constant and, in particular, considerable hydrogen-bonding abilities, which has been suggested to be the main prerequisite for surfactant self-assembly [1].

The term solvophobicity, the use of which is currently widespread in the field of the association colloids, refers to the limited affinity of a certain species for the solvent in which it finds itself [3]. Indeed, the well-studied hydrophobic interaction is not exclusive to water but is a particular case, perhaps the most important, of a more general solvophobic interaction [4]. In light of the decisive role of the so-called hydrophobic effect in the micellization process, there is an increasing interest in broadening this understanding to the case of more complex solvent systems. A reasonable and common approach comprises partially replacing water with another polar organic solvent, process that allows a wide range of polarities and solvophobicities to be assessed. As a result, numerous studies with both ionic and nonionic surfactants have been conducted in the last decades using this experimental strategy [5–47]. Among them, the recent contributions of a few groups deserve, in our opinion, special comment. For example, Moyá and co-workers [16–24] have paid particular attention to the characterization of different micellar systems in several mixed aqueous-organic solvents. These authors have both studied the micellar properties of these surfactant systems and have evaluated them as reaction media [16,19,23]. Moreover, it is important to point out the contributions of this group to correlating changes in the Gibbs energy of micellization with the Gordon parameter, which describes the bulk phase cohesive energy density [20,24]. Similar studies by Alexandridis and co-workers in the field of block copolymers are also relevant [25–31]. These researchers have presented important findings in aspects such as the effect of the co-solvent on the structure and microstructure of the aggregates formed in the mixed media. Eastoe and co-workers have recently investigated the adsorption behavior and structural changes in micelles of nonionic surfactants formed in mixed glycol media [32–34]. These authors noted the ability of these solvent systems to control surfactant aggregation, which is important when it comes to broadening their possible applications. Our laboratory has also an extensive track record in this field [35–42], and we have studied the effect of ethylene glycol, formamide and glycerol on the micelle formation of ionic and nonionic surfactants. Our interest has centered on the characterizing the interactions responsible for the micellization process in these solvent systems and their effects on the structure and the microenvironmental properties of the aggregates formed in them. However, judging by the number and relevance of publications that continue to appear in this field [43–47], the current interest in micelle formation in polar organic solvents and their aqueous mixtures is evident.

Among the many amphiphilic materials available, nonionic surfactants are often preferred in many biochemical and pharmaceutical applications as they show a greater tolerance to changes in pH or to the presence of electrolytes and also because they typically present a favorable behavior in the stabilization of proteins and exhibit a reduced toxicity profile [48]. Alkylglucosides form the main group of so-called sugar-based surfactants, a relatively new class of nonionic surfactants that are receiving increased attention due to their advantages in terms of consumer health, environmental compatibility, and performance compared to other conventional nonionic surfactants [49–52]. In particular, as a result of their unique properties, alkylglucosides are frequently used in the solubilization and stabilization of membrane proteins [53].

**Figure 1:**
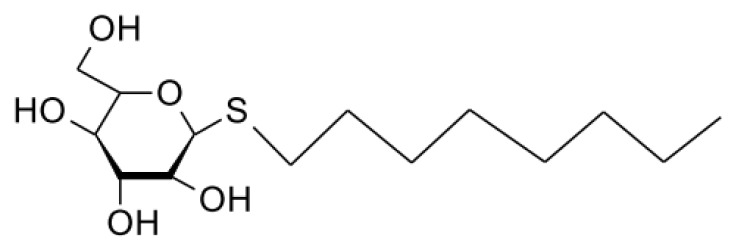
Molecular structure of *n*-octyl-β-d-thioglucopyranoside (OTG).

Herein we present a study of the effect of ethylene glycol on micellization of the alkylglucoside surfactant *n*-octyl-β-d-thioglucopyranoside (OTG). We have chosen this surfactant for two reasons: first, because it is a representative member of the alkylglucoside group, whose advantages in the membrane protein field have been demonstrated [54–57], and second, to take advantage of our understanding of its aggregation behavior in the pure state and in mixtures with other ionic and nonionic surfactants, which we have previously studied in detail [58–64]. The molecular structure of OTG is depicted in Figure 1, where it can be seen that OTG is mainly characterized by a bulky and rigid headgroup, which results in differences in the hydration and packing behaviors with respect to those of conventional ethoxylated nonionic surfactants.

This paper is presented in three parts. Firstly, we examine the effect of EG addition on the micellization of OTG using surface tension measurements and the pyrene 1:3 ratio method. In addition, the surface tension isotherms obtained are used to analyze the adsorption behavior at the air-liquid interface. The second part deals with a combined static and dynamic light scattering study to obtain information on the size of the aggregates formed in the different solvent systems. Finally, we attempt to characterize the alterations in the micellar microstructure induced by the solvent composition from the rotational behavior of a hydrophobic probe (C153) solubilized in micelles.

## 2. Results and Discussion

### 2.1. Micellization and Adsorption Properties

**Figure 2:**
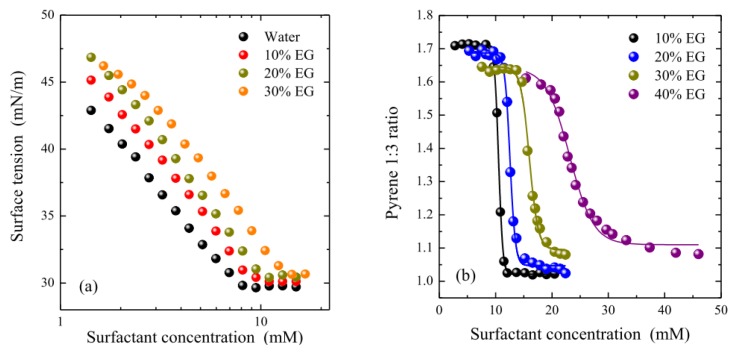
(**a**) Surface tension isotherms of OTG and (**b**) plots of the pyrene 1:3 ratio index as a function of surfactant concentration in several solvent systems whit different EG contents at 25 °C. The adsorption isotherm in 40% EG and the plot of the pyrene 1:3 ratio index in pure water were omitted for the sake of clarity.

To examine the effect of the co-solvent on the interfacial behavior of OTG, the surface tension of surfactant solutions was recorded as a function of their concentration in a number of solvent systems with different EG contents. It can be seen from Figure 2a, which shows the results of these experiments, that the surface tension behaves in a similar manner to that of pure water in all cases, in other words, it decreases with increasing surfactant concentration then remaining nearly constant above a certain concentration. The cmcs, which are determined from the breakpoint of the two parts of the isotherm, are reported in Table 1. Cmc values were also obtained using the pyrene 1:3 ratio method [65]. Figure 2b shows the plots of the pyrene 1:3 ratio index as a function of surfactant concentration for different solvent systems. The cmcs were obtained from these plots, which show a typical sigmoidal decrease, using the data treatment previously developed in our laboratory [66]. The cmc values obtained using this latter method are also listed in Table 1. Although both sets of results present a similar tendency, it can be seen that the pyrene 1:3 ratio method provides systematically higher values than those obtained from surface tension measurements. This discrepancy is due to the different experimental techniques used. All probe-based methods systematically report higher cmc values as they require the formation of aggregates in which the probe can be incorporated. It should be noted that we found an acceptable reproducibility of the experimental results with both techniques in all cases except for the system containing 40% EG, possibly because micellization of OTG is strongly inhibited in this solvent system.

Table 1 also lists the values of the Gibbs energy of micellization Δ*G*_mic_^0^, as calculated by

(1) $$\Delta G_{\text{mic}}^{0}=RT\;\text{ln cmc}$$

where *R* and *T* have their usual meaning and the cmc values are expressed in the molar fraction scale. It can clearly be seen from Table 1 that the addition of EG makes the Δ*G*_mic_^0^ values less negative, thereby suggesting that the micellization ability of OTG weakens as the content of co-solvent increases. The micellization of nonionic surfactants in EG-water mixtures is essentially controlled by two factors, namely the structure-breaking ability of the co-solvent and its interaction with the surfactant headgroups. Our cmc and Δ*G*_mic_^0^ data (Table 1) reflect the fact that the co-solvent is acting as a structure breaking agent, thereby decreasing the solvophobic effect and favoring the solubility of OTG monomers in the water-organic solvents.

**Figure 3:**
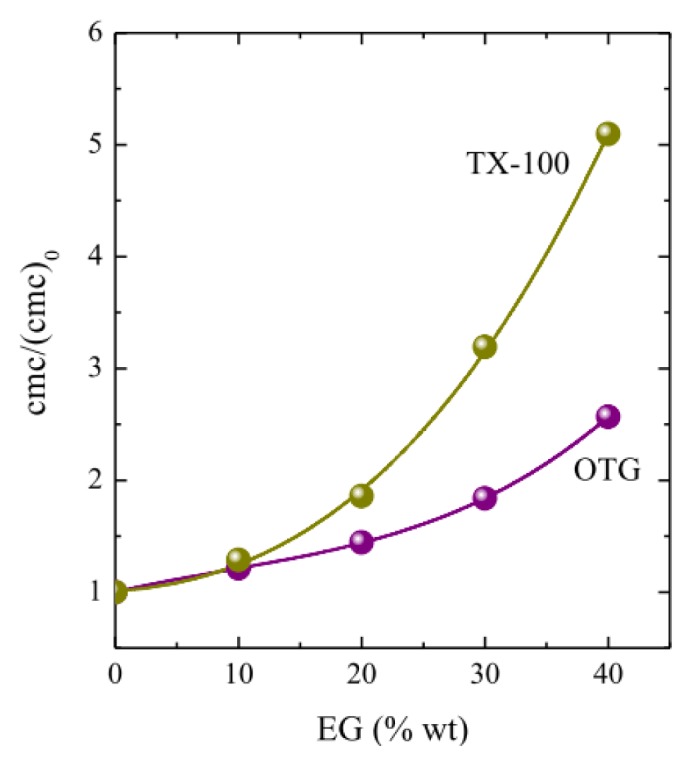
Effect of EG addition on the relative cmc values of OTG and TX-100. (cmc)_0_ is the cmc in pure water. Data plotted are the cmc values obtained by the pyrene 1:3 ratio method.

In the context of our investigation, it is illustrative to compare the effect of EG addition on the micellization of OTG with that of a conventional ethylene oxide-based surfactant, namely *p*-tert-octyl-phenoxy polyethylene (9.5) ether (Triton X-100 or TX-100), a representative member of that family whose aggregation behavior in EG-water mixtures has been studied by us [37,66]. Figure 3 shows the relative increase of the cmc with respect to the cmc values in pure water, (cmc)_0_, for OTG and TX-100.

The data in Figure 3 indicate that the cmc of TX-100 is more strongly affected by addition of the co-solvent than that of OTG. The main difference between these two surfactants resides in the structure of their headgroups: flexible and polymer-like for TX-100 and rigid and bulky for OTG. These differences determine both the hydration behavior of these two surfactants and the way in which they pack when monomers absorb at the air-liquid interface or self-assemble to form the micelle. In fact, it has been established that although ethoxylated surfactants are more hydrated than alkylglucosides, water is more strongly bound to the sugar heads [67,68]. This means that the H-bonds between the sugar head and surrounding water molecules are more difficult to break than in the case of polyoxyethylene (POE) groups, thus meaning that the number of water molecules that interact with the headgroups of OTG via hydrogen bonding is more weakly affected.

**Figure 4:**
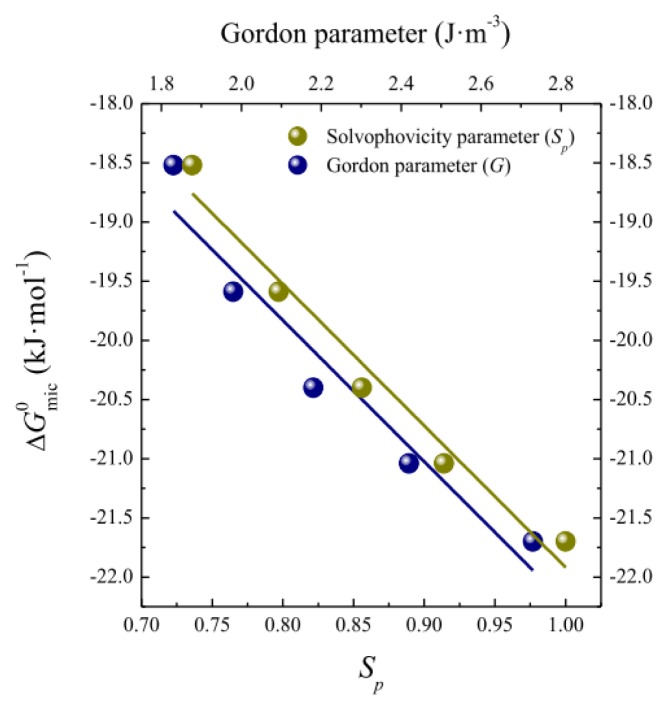
Plots of the Gibbs energy of micellization Δ*G*_mic_^0^, as a function of the solvophobic parameter, *S_p_*, and of the Gordon parameter, *G*, in various EG-water solvent mixtures.

In accordance with the observations of Moyá and co-workers [20,24], we also attempted to correlate the Gibbs energy of micellization for OTG with solvophobic parameters. Initially, we used the Gordon parameter, *G*, which gives a measure of the cohesiveness of the solvent and therefore provides an indication of the driving force for micellization. The Gordon parameter *G* is given by *G* = γ/*V*_m_^1/3^ where γ is the air-liquid surface tension and *V*_m_ the molar volume of the solvent. We also employed a solvophobic parameter, *S_p_*, which characterizes the interactions of alkyl chain with solvents [3,69] and which has also been shown to correlate well with the Gibbs energy of aggregation of ionic liquids [44]. Using calculated and literature values for *G* and *S_p_* [44], respectively, for EG-water mixtures, we plotted the Gibbs energy of micellization for OTG as a function of both parameters in Figure 4.

It can clearly be seen from Figure 4 that the reduced values of the Gordon and solvophobic parameters induced by EG addition make the Gibbs energy values less negative, and hence the micellization of OTG less spontaneous. Furthermore, it can also be seen that the decrease of Δ*G*_mic_^0^ with *G* and *S_p_* is acceptably described by a linear function, as has also been observed for other surfactants and solvent mixtures [20,24,44]. These observations can be interpreted by considering that the micellization ability of OTG is weakened as the EG content in the solvent system increases. The presence of EG reduces the solvophobic effect and, as a consequence, micelle formation becomes less favorable.

The adsorption parameters of the surfactant in the different solvent mixtures were derived from the equilibrium surface tension isotherms in Figure 2a. The surface excess concentration, Γ_max_, represents a measure of the effectiveness of surfactant adsorption, and can be determined for dilute solutions from the Gibbs adsorption Equation [70],

(2) $$\Gamma_{\text{max}}=-\frac{1}{2.303\;RT}{\left[\frac{\partial \gamma }{\partial \text{log }c}\right]}_{T,P}$$

where γ is the corrected surface tension and *c* the surfactant concentration. The minimum area per surfactant molecules *A*_min_, at the air-liquid interface can be obtained from the Γ_max_ values using the Equation

(3) $$A_{\text{min}}=\frac{1}{N_{\text{A}}\;\Gamma_{\text{max}}}$$

where *N*_A_ is Avogadro’s number. On the other hand, the effectiveness of a surface-active molecule is given by the surface pressure at the cmc Π_cmc_, which can be determined by the relationship

(4) $$\Pi_{\text{cmc}}=\gamma_{0}-\gamma_{\text{cmc}}$$

where γ_0_ is the surface tension of the pure solvent and γ_cmc_ the surface tension of the solution at the cmc, that is, when the air-liquid interface is fully saturated with surfactants. Finally, the standard free energy of adsorption Δ*G*_ads_^0^, was calculated using the Equation [70]

(5) $$\Delta G_{\text{ads}}^{0}=\Delta G_{\text{mic}}^{0}-\frac{\Pi_{\text{cmc}}}{\Gamma_{\text{max}}}$$

where the standard state for the surface phase is defined as a hypothetical surface covered with a monolayer of surfactant at its closest packing but at a surface pressure equal to zero [70].

The values obtained for these adsorption parameters are listed in Table 1, where it can be seen that Γ_max_ increases upon EG addition. This behavior is opposite to that observed by us for TX-100 [37] and Tween 20 [40], but is similar to the initial trend of this parameter for OTG upon addition of NaCl [59]. In this case, we attributed the aforementioned increase in Γ_max_ to dehydration of the hydrophilic group induced by the presence of the additive. This interpretation would also be consistent with a reduction in *A*_min_ with increasing EG content. Moreover, it can be also concluded from the high values of *A*_min_ in Table 1 that the hydrophobic chains adsorbed at the air-liquid interface are not in a close-packed arrangement normal to the interface at saturation adsorption. This is probably due to the formation of surface clusters of monomers, as previously observed for different sugar-based surfactants [71]. The data in Table 1 also indicate that the standard free energy of adsorption, Δ*G*_ads_^0^, is negative in all cases but becomes less negative upon EG addition. Furthermore, the Δ*G*_ads_^0^ values are more negative than their corresponding Δ*G*_mic_^0^ values, thus indicating that adsorption of the surfactant at the air-liquid interface is more favorable than micelle formation, a situation which is clearly affected by the presence of co-solvent in the bulk phase.

With regards to the effect of EG on the surface activity of other non-ionic surfactants, it has already been mentioned that our results for OTG are in contrast to those obtained for TX-100 [37] and Tween 20 [40]. Similarly, although Seguin *et al*. [32] have studied the effect of EG on the interfacial behavior of the non-ionic surfactant octaethylene glycol monododecyl ether (C_12_E_8_), a comparison with their data is difficult because these authors only used three solvent systems, namely pure water, water-EG (50%) and pure EG. Thus, Γ_max_ is observed to increase for the mixed solvent and then to decrease for pure EG. The reverse tendency is, of course, observed for *A*_min_. It should also be mentioned that, according to previous observations [71], the surface activity of ethoxylated-based surfactants exhibits some clear differences compared to sugar-based ones. It seems that these differences are a consequence of the different nature of the headgroups of both kinds of surfactants, which cause differences between the surface packing of monomers at the air-liquid interface as well as in the structure of water around their hydrophilic groups. As such, it is not surprising to find different interfacial behaviors with the addition of EG.

### 2.2. Micellar Size

**Figure 5:**
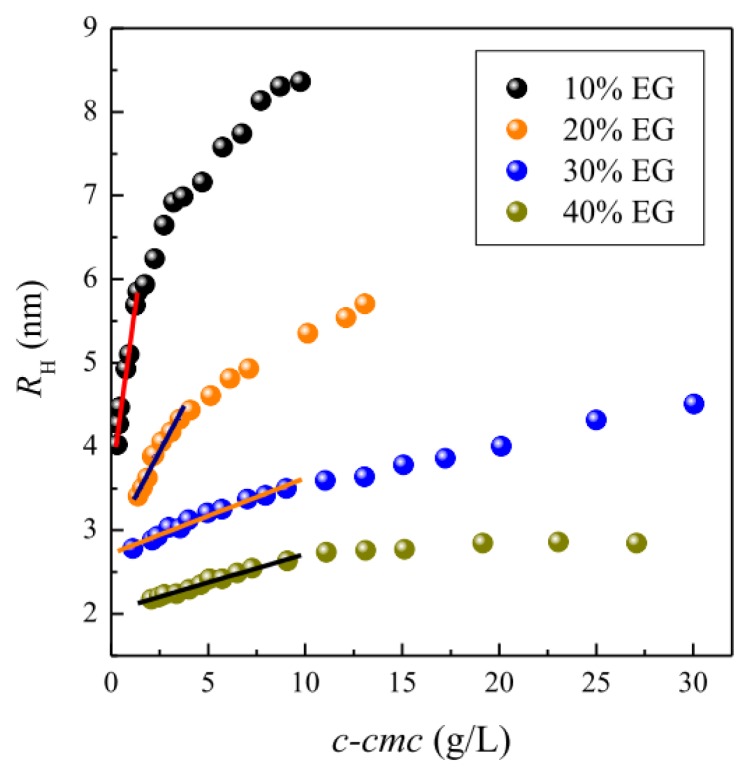
Apparent hydrodynamic radius of micelles, *R*_H_, as a function of the micellar concentration in different water-EG solvent mixtures. The solid lines are the best linear fit to the experimental data.

Both static and dynamic light scattering studies were carried out to examine the influence of EG addition on the micellar size of OTG. The dynamic light scattering (DLS) experiments showed a monomodal distribution peak with relatively narrow size distribution in all cases. This indicates that no undefined aggregation occurs, but rather that these single scattering entities can be identified as the micelles of OTG in the different water-EG solvent mixtures. Figure 5 displays the apparent hydrodynamic radius of the micelles, *R*_H_, as a function of the micellar concentration at different EG percentages (10%–40%). As can be seen from this figure, *R*_H_ increases with the micellar concentration. A general trend observed in this figure is the existence of two regions: a surfactant concentration range in which the micellar size depends linearly on the micellar concentration, and a second region, above a certain surfactant concentration, where *R*_H_ ceases to be linear in *c-cmc*. This latter trend suggests micellar growth at elevated surfactant concentrations. Analysis of these data in the low concentration range, where the experimental results are well described by Equation 17 (see Experimental section), allows the hydrodynamic radius *R*_0_ and the coefficient *k_D_*, which are listed in Table 2, to be derived by extrapolation at infinite dilution. The data in Figure 5 clearly indicate that OTG micelles become smaller in size as the EG content increases.

Taken together, the data in Figure 5 and Table 2 allows us to conclude that the concentration dependence of *R*_H_ is affected by the *k*_D_ coefficient but that this dependence is less pronounced (smaller values of *k*_D_) as the EG content in the solvent system increases. Similar results were obtained by us in the case of ethoxylated surfactants (Triton X-100 and Tween 20) [37,40]. Table 2 shows that the *k*_D_ values are negative and strongly dependent on the EG content in the bulk phase. The parameter *k*_D_ is connected to the intermicellar interaction potential. Generally, a larger attractive interaction leads to a more negative virial coefficient [72], thus meaning that the attractive interactions between micelles are gradually reduced upon addition of EG, and are practically negligible for solutions containing 30% and 40% EG.

**Figure 6:**
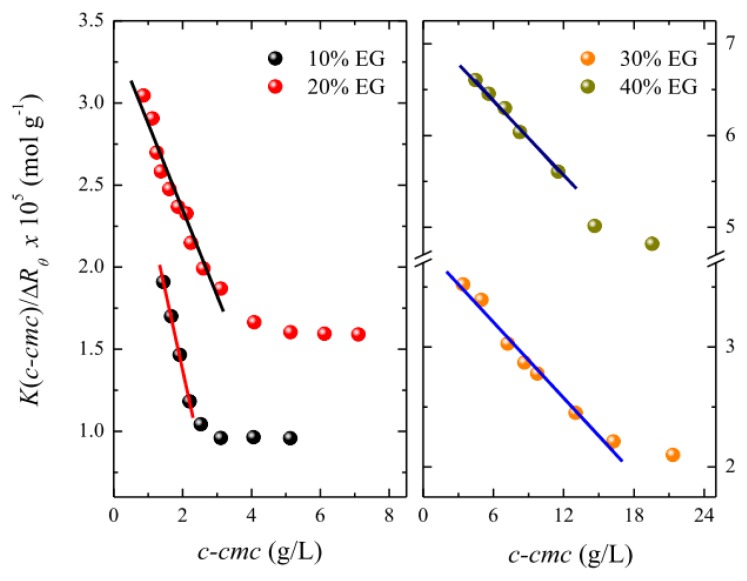
Debye plots for OTG micellar solutions in different water-EG solvent mixtures. The solid lines are the best linear fit to the experimental data according to Equation (14) (see Experimental section).

On the other hand, static light scattering (SLS) measurements were performed to obtain the molecular weight of the micelles, *M*_W_, and, hence, the micellar aggregation number, *N*_agg_. Figure 6 shows typical Debye plots for surfactant solutions in various solvent systems.

The Debye plots in Figure 6 decrease linearly as a function of the micelle concentration but deviate downward at a certain concentration. The molecular weight of micelles, *M*_W_, and the second osmotic virial coefficient, *A*_2_, were obtained from the intercept and slope, respectively, of the linear region, where micellar growth is negligible. The values of *A*_2_ together with those of the mean aggregation numbers, *N*_agg_, are also listed in Table 2. It is important to note here that the *N*_agg_ value obtained for OTG in pure water is in excellent agreement with that reported previously by Frindi *et al.* [73] using time-resolved fluorescence measurements. As can be seen, an increase in the amount of organic solvent present in the micellar solutions produces a decrease in the aggregation number. In the solution with 40% EG, the aggregation number of the micelles decreases by around 60% in comparison with pure water, whereas the corresponding hydrodynamic volume is reduced by 80%. This fact could suggest that the modification in micellar solvation plays a relevant role in the reduction of the micellar dimensions upon addition of the organic solvent.

The second osmotic virial coefficient can be used as an indication of intermicellar interactions in solution. Like the coefficient *k*_D_, *A*_2_ also has small and negative values, thus indicating that a weak attractive interaction exists between the micelles. We also observed a systematic decrease in *A*_2_ with increasing EG content in the EG-water mixture, thus indicating that intermicellar interactions become less attractive at higher EG concentrations. The presence of EG molecules in the solution affects the interactions between both the hydrophobic and the hydrophilic groups in the surfactants. The dielectric constant of EG is much lower than that of water. As such, the reduction in *A*_2_ as EG is added to the aqueous solution could be explained by a reduction of the attractive van der Waals interactions as the addition of EG to water reduces the dielectric constant of the mixed solvent system [74,75]. It should be noted at this point that the effects of intermicellar interaction on *A*_2_ are similar to those on *k*_D_, that is, both parameters decrease in the same way with increasing EG content.

Some authors have reported that the second osmotic virial coefficient obtained by SLS measurements describes the thermodynamic quality of the solution, which is defined as good when solute-solvent interactions are preferred over solute-solute interactions (*A*_2_ > 0) and poor when solute-solute interactions are favored over solute-solvent interactions (*A*_2_ < 0) [76–79]. In the case of micelles, *A*_2_ is a measure of the surfactant-solvent interaction in solution and its value denotes the strength of such interactions [80]. The values of the *A*_2_ parameter collected in Table 2 suggest that surfactant-surfactant interactions are less favored with increasing EG content, thus indicating a lower tendency of OTG to form micelles. The fact that the *A*_2_ values are small and negative indicates that the surfactant-surfactant attractions are weak but dominant. It is interesting to note that the different media used in our experiments are poor solvents for OTG, but that the water-EG mixtures are better solvents for the surfactant than pure water. This behavior arises due to the structure-breaking nature of EG and the ability of the co-solvent to reduce the solvophobic effect, as discussed previously. In addition, we should not forget that the addition of EG to the solvent system decreases the dielectric constant with respect to pure water, thereby helping to reduce the inter-surfactant interactions. OTG molecules easily form large aggregates in water, whereas they tend to form micelles with a smaller size in 40% water-EG solutions. Larger and negative *A*_2_ values, thus meaning a strong and attractive inter-surfactant interaction, result in the formation of micelles with larger aggregation numbers [81].

The second virial coefficient for the micelles can be expressed in terms of a sphere volume (the so-called effective volume, *V*_EFF_) that is equivalent to a hard and homogeneous sphere. The value of the effective volume accounts for the micelle interactions, and its value for particles interacting as hard spheres is equal to the micelle volume, *V*_S_. In contrast, *V*_EFF_ may be substantially different from *V*_S_ if long-range interactions are present. *V*_EFF_ can be related to the second virial coefficient using the Equation [58,59,82]:

(6) $$V_{\text{EFF}}=\frac{1}{4}\frac{A_{2}\;M_{\text{W}}^{2}}{N_{\text{A}}}$$

Further, we estimated the effective radius of the micelles, *R*_EFF_, as a function of EG concentration from *V*_EFF_. The calculated values are presented in Table 2. In the absence of long-range repulsive forces, as would occur for non-ionic surfactants, the attractive van der Waals forces dominate the long-range interaction. The results obtained imply that these interactions are attenuated by the presence of EG in the solvent, probably as a result of a decrease in the dielectric constant of the medium.

In addition, light scattering experiments allowed us to obtain information about the average density, ρ, of the OTG micelles. This parameter was calculated from *M*_W_ and *R*_0_ according to the following Equation [76,78,80]:

(7) $$\rho =\frac{3\;M_{\text{W}}}{4\;\pi \;N_{\text{A}}R_{0}^{3}}$$

The ρ values so obtained are reported in Table 2, from which it can be seen that ρ increases with EG content. This effect is likely due to the fact that addition of EG to the bulk phase causes a reduction in the amount of water bound to the micelles and therefore an increase in their density. In other words, the presence of the co-solvent induces micellar dehydration, thus leading to a less permeated structure.

### 2.3. Micellar Microstructure

We studied the photophysics and dynamics of the hydrophobic probe coumarin 153 (C153) solubilized in the micellar pseudophase with the aim of examining how EG addition affects the local structure of the OTG micelles. Coumarin 153 is an extensively used probe whose notable photophysical properties have been widely exploited to investigate the micropolarity and microviscosity of several nanoscopic environments, including supramolecular host cavities, micelles, polymers, *etc.* [83]. Indeed, we have recently used this probe to assess the microenvironmental properties of mixed nonionic surfactant systems consisting of a sugar-based and an ethoxylated surfactant [63,84].

**Figure 7:**
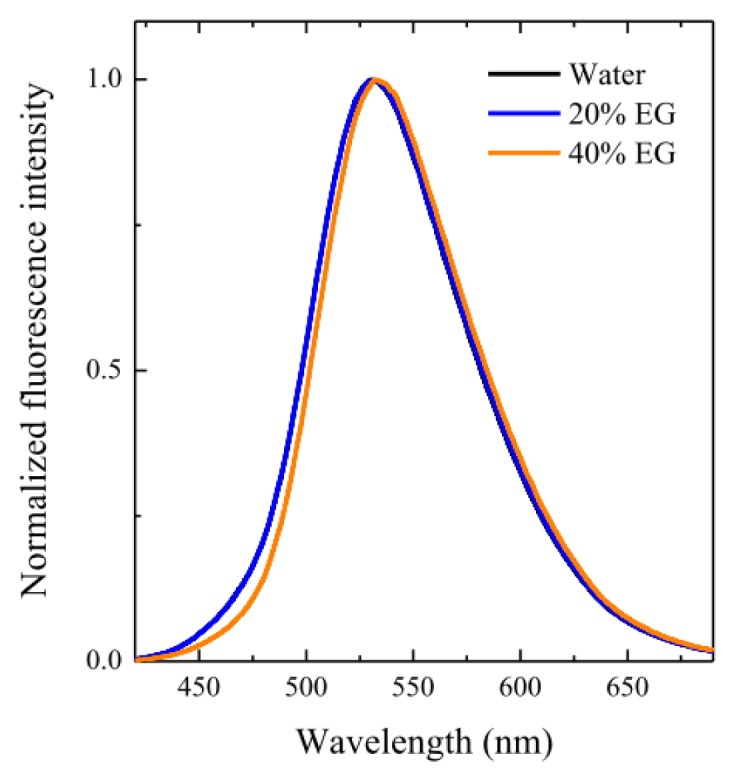
Steady-state emission spectra of C153 in OTG micellar solutions (30 mM) at different water-EG solvent mixtures (λ_exc_ = 405 nm) and 25 °C. The spectrum in water is plotted but matched by that in 20% EG. The spectra in 10% and 30% EG are omitted for the sake of clarity.

Initially, we performed a spectroscopic study of C153 in micellar media at different solvent compositions. It can be seen from Figure 7, which shows the fluorescence emission spectra of the probe in OTG micelles formed in various solvent systems, that the steady-state fluorescence spectra of C153 consists of a broad band centered around 532 nm. It is important to note that the spectrum in pure water is essentially identical to those in 10%, 20% and 30% EG, with a very small red-shift of about 1 or 2 nm only being observed when the EG content reaches 40%. These minor variations, together with the observed reduction in the micellar size, make it difficult to draw conclusions regarding changes in the local polarity around the probe.

**Figure 8:**
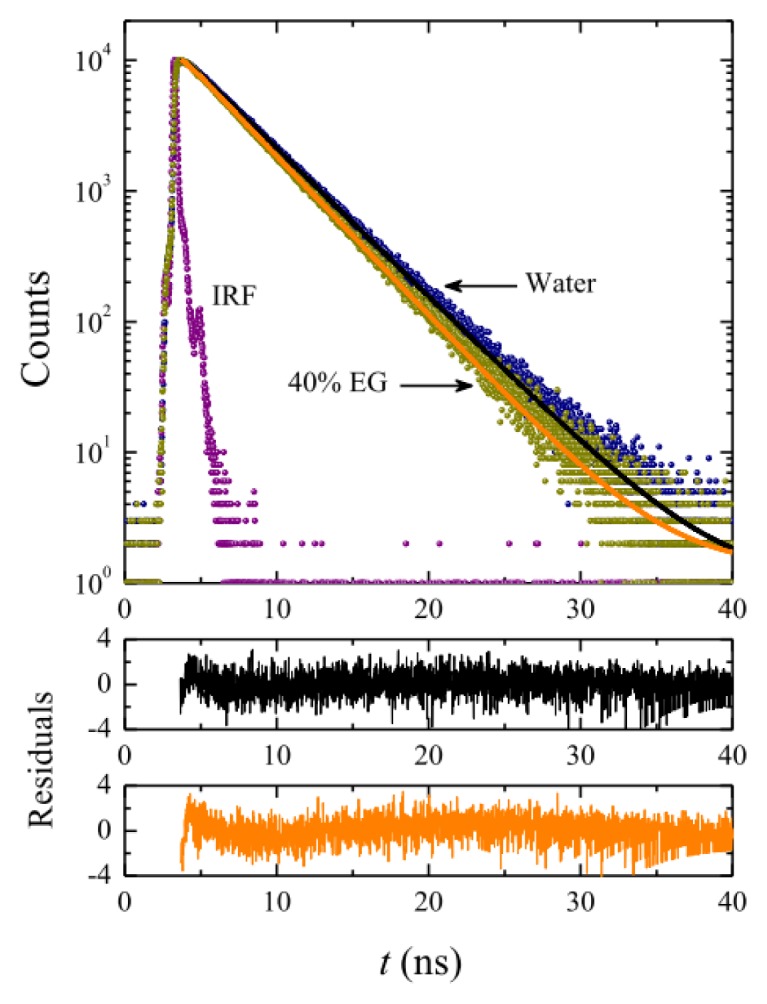
Fluorescence decay of C153 in OTG micellar solutions (30 mM) in pure water and in a solvent system with 40% EG at 25 °C. The solid lines through the data points are the best fit to a single-exponential function. The corresponding weighted residuals are also shown. IRF is the instrumental response function.

Figure 8 shows representative fluorescence decays of C153 solubilized in OTG micelles. It should be noted that, judging from the χ^2^ values (≤1.15) and the corresponding residuals, all the fluorescence decays of C153 are well-fitted with a single-exponential function. The values of the fluorescence lifetimes of the probe were 3.83 ns in pure water, 3.82 ns in 10% EG, 3.79 ns in 20% EG, 3.71 ns in 30% EG, and 3.56 in 40% EG. These values are similar to those obtained in methanol (4.02 ns) or ethanol (3.40 ns), thus suggesting that the probe probably resides in the palisade layer close to the micelle-water interface. Our data indicate that the lifetime values decrease slightly but systematically with increasing content of the co-solvent. However, due to the dramatic reduction in the micellar size upon EG addition, it is also difficult to draw conclusions from this tendency.

**Figure 9:**
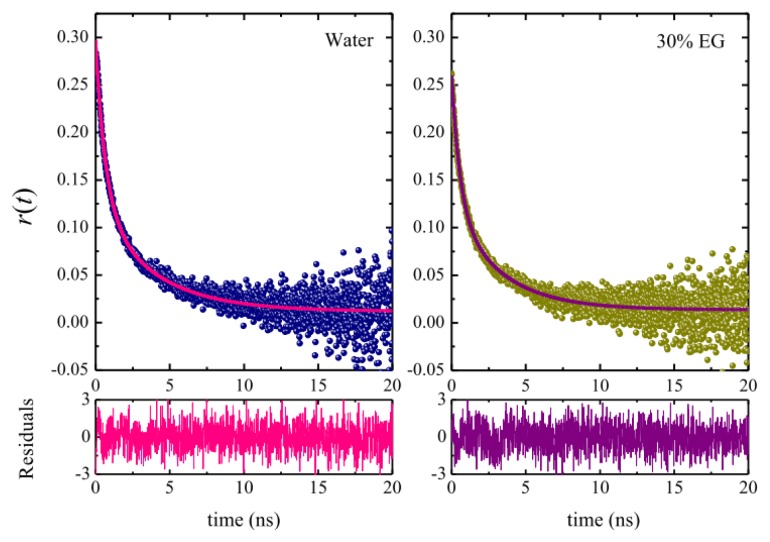
Fluorescence anisotropy decays of C153 in OTG micellar solutions (30 mM) in pure water and in a solvent system with 30% EG at 25 °C. The solid lines through the data points are the best fit to a biexponential function. The corresponding weighted residuals are also shown.

Time-resolved fluorescence anisotropy studies were carried out to obtain further insight into the micellar microenvironment around C153 as a function of the solvent composition. This technique is considered to be a very sensitive way to determine the rotational relaxation of a molecular probe in restricted environments [85]. Figure 9 shows some examples of the fluorescence anisotropy decays obtained in OTG micellar systems in pure water and in 30% EG. Similar decay curves were observed in all the other media. Anisotropy decay of probes attached to micelles is often analyzed by the so-called two-step model [86,87], which is characterized by two time constants according to the Equation [85],

(8) $$r(t)=r_{0}\left[\beta \;\text{exp}\left(-\frac{t}{\tau_{\text{slow}}}\right)+(1-\beta )\text{exp}\left(-\frac{t}{\tau_{\text{fast}}}\right)\right]$$

where τ_slow_ and τ_fast_ are the two reorientation times of the probe in the micelle, *r*_0_ is the anisotropy at time zero, and β is a pre-exponential factor giving the relative contributions of the slow, τ_slow_, and fast, τ_fast_, time constants. The anisotropy decay parameters obtained upon fitting our decay curves to Equation (8) are given in Table 3.

With regard to the biexponential character of the anisotropy decays of the probe observed in our micellar systems, it has been well established that this behavior is not due to the location of the probe in two distinct regions of the micelle [86], as evidenced by the fact that fluorescence decays follow a single-exponential function. Rather, the observed biexponential anisotropy decay is due to different kinds of motion experienced by the probe. The two-step model establishes that, depending on its position, the probe undergoes slow lateral diffusion at or near the micellar interface as well as fast wobbling motion in the micelle, with both these motions being coupled to the rotation of the micelle as a whole [86–89].

The data in Table 3, and the fitting results in Figure 9, indicate that the anisotropy decay curves of C153 in our micellar systems are acceptably fitted by Equation (8). In addition, Table 3 also lists the average reorientational times, 〈τ*_r_*〉, for all the systems studied, which were calculated using the Equation

(9) $$\langle \tau_{r}\rangle =\beta \;\tau_{\text{slow}}+(1-\beta )\;\tau_{\text{fast}}$$

The data in Table 3 also indicate that, although it initially remains constant, the average reorientational time subsequently decreases as the EG content in the solvent system increases. Moreover, we were able to calculate the so-called generalized order parameter, *S*, using the Equation *S*^2^ = β [86], which provides information regarding the motional restriction of the probe in the micelle. This parameter is a measure of the equilibrium orientational distribution of the probe and has values ranging from 0, for unrestricted motion, to 1 for completely restricted motion. Since the β parameters obtained herein are essentially constant (see Table 3), the corresponding *S* values (around 0.64) are also constant. This finding has two implications. First, the value of 0.64 is considered a high value and hence can be taken to indicate that the probe is located in the micellar palisade layer, close to the interface, where a high degree of order is expected [89]. Second, since the *S* parameter remains constant upon EG addition, it can be inferred that the probe does not modify its localization in the micelle.

In order to gain a better understanding of the rotational dynamics of the probe molecule in our micellar systems, we analyzed our experimental data using the Stokes-Einstein-Debye (SED) hydrodynamic model of rotational diffusion. According SED theory, the rotational motion of a medium sized solute molecule in a solvent continuum is assumed to occur by small step diffusion, and its reorientation time is related to the solvent viscosity and temperature. In the case of a noninteracting solute in a medium of viscosity η, in our case the local viscosity around the probe or micellar microviscosity, η_m_, the reorientational time 〈τ*_r_*〉 is given by

(10) $$\langle \tau_{r}\rangle =\frac{V_{\text{H}}\;\eta_{\text{m}}}{k_{\text{B}}\;T}$$

where *V*_H_ is the hydrodynamic volume of the solute and *k*_B_ and *T* are the Boltzmann constant and absolute temperature, respectively. However, the application of Equation (10) requires some considerations. First of all, the experimentally obtained 〈τ*_r_*〉 values have two contributions: the rotation of the probe in the micelle and the rotation of the micelle itself [89]. The contribution of the second rotational motion is decisive when the micelle size is small and its reorientational time is comparable to 〈τ*_r_*〉. As the size of the OTG micelles is strongly affected by EG addition, it is important to evaluate the effect of the rotational motion of the micelle on the reorientational time of the probe in the micelle. Thus, the average reorientation time of the probe in the micelles, 〈τ*_r_*〉_P_, was obtained using the following relationship [89].

(11) $$\frac{1}{\langle \tau_{r}\rangle }=\frac{1}{{\langle \tau_{r}\rangle }_{\text{P}}}+\frac{1}{\tau_{\text{M}}}$$

where τ_M_ is the time constant for the overall rotation of the micelle, which can also be evaluated, according to the SED hydrodynamic theory, using the Equation

(12) $$\tau_{\text{M}}=\frac{V_{\text{M}}\;\eta_{\text{sol}}}{k_{\text{B}}\;T}$$

where *V*_M_ is the hydrodynamic radius of the micelle, η_sol_ is the viscosity of the bulk phase, and *k*_B_ and *T* have their usual meaning. To calculate *V*_M_, we obtained the apparent hydrodynamic radius of OTG micelles at the surfactant concentration used in the spectroscopic experiments (30 mM). The *V*_M_ results obtained are listed in Table 4, together with those of 〈τ*_r_*〉_P_ from Equation (11). It is clear from these data that the micellar size only affects the average reorientational times of the probe in the systems with high EG content (30% and 40%).

On the other hand, the application of Equation (10) requires an estimation of the hydrodynamic volume of the probe, which is a product of the van der Waals volume, *V*, shape factor, *f*, and the boundary condition parameter, *C*_slip_, that is, *V*_H_ = *VfC*_slip_ [89]. In this way, the microviscosity of micelles can be estimated as

(13) $$\eta_{\text{m}}=\frac{{\langle \tau_{r}\rangle }_{\text{P}}\;k_{B}\;T}{V\;f\;C_{\text{slip}}}$$

Adopting the literature values for C153 [90,91] of 246 Å^3^, 1.71 and 0.57 for *V*, *f* and *C*_slip_, respectively, we obtained the microviscosity values listed in Table 4. The η_m_ value obtained for OTG micelles in pure water is much higher than that found for ionic micelles, but only marginally higher than that obtained for TX-100 (27.4 mPa s) [92]. This fact reflects a more structured palisade layer of OTG as a result of the bulkier and rigid headgroup of OTG compared to that of TX-100. On the other hand, it can be seen that η_m_ remains almost constant for those systems with a low EG content, but decreases somewhat when the EG content reaches 30% or 40%. On the other hand, it is important to note that the behavior of OTG is opposite to that previously observed by us for TX-100 (37) and Tween 20 (40). In these cases, using a similar neutral probe (coumarin 6), it was observed an increase in the micellar microviscosity with the addition of EG, which was ascribed to the participation of EG in the solvation of the headgroups of the surfactants. On the contrary, the reduction in the micellar microviscosity observed for OTG in the high concentration regime of the co-solvent (see Table 4), indicating a decrease in the rigidity around the probe, must be interpreted in the sense that EG does not penetrate into the solvation layer of the micelles, but rather as a result of a certain dehydration in this micellar region. We postulate that this different behavior is likely due to the lower affinity of EG toward the sugar headgroups of OTG in relation to those of ethoxylated surfactants, and also to the fact that water molecules are more strongly bound to sugar groups and, consequently, it is much more difficult to remove just a few water molecules and replace them with some of EG.

## 3. Experimental Section

### 3.1. Materials

The surfactant *n*-octyl-β-d-thioglucopyranoside (OTG) was supplied by Sigma and used without further purification. Ethylene glycol (99%+, spectrophotometric grade) was purchased from Aldrich (Munich, Germany). The fluorescence probes pyrene (Py) from Sigma (Munich, Germany) and coumarin 153 (C153) from Exciton (Dayton, OH, USA) (laser grade) were used as received. Stock solutions of the surfactant in pure water and in water-EG mixtures were prepared by dissolving a known mass in the solvent system. Working solutions were prepared daily by diluting the stock solutions. The ultrapure water (resistivity: ~18 MΩ cm) used to prepare the micellar solutions was obtained by passing pure water from a Millipore Elix system through an ultra-high quality Millipore Synergy purification system. Two 1 mM stock solutions of the fluorescence probes were prepared in methanol and stored at 4 °C. Measurement samples of 1–2 μM in Py and 7 μM in C153 were prepared by adding small volumes of the methanolic solutions to the respective micellar solutions.

### 3.2. Methods

#### 3.2.1. Surface Tension

Air-liquid equilibrium surface tension measurements were performed using the Du Noüy ring method, with a Sigma 701 (KSV) tensiometer, following the previously described procedure [58]. Briefly, each series of measurements was started with a concentrated solution of surfactant, and successive diluted solutions were obtained by adding either pure water or EG-water solvent mixtures to a jacketed vessel whose temperature was maintained at 25 °C using a circulating water bath. After each dilution, the resultant surfactant solution was stirred and then stabilized for at least 60 min before carrying out the measurement. The surface tension values were accurate to within ±0.1 mN m^−1^. The reproducibility of the cmc was found to be less than ±15.0% (standard deviation of the mean) calculated from the experimental cmc data of at least two runs.

#### 3.2.2. Light Scattering

All light scattering measurements were obtained using a Zetasizer Nano-S system (Malvern Instruments, Worcestershire, UK). This apparatus, which uses a backscattering detection system (scattering angle θ = 173°), is fitted with a Helium-Neon laser source (632.8 nm and 4.0 mW) and has a built-in Peltier temperature control with an accuracy of ±0.1 °C. Micellar solutions of varying concentration were prepared in different solvent systems. These solutions were filtered directly into the cuvettes using membrane filters with a pore size of 0.1 μm. The cuvette was rinsed several times with ultrapure water prior to each measurement and then filled with filtered micellar solutions. The apparent hydrodynamic radius of the micelles, *R_H_*, was obtained using dynamic light scattering measurements, which were analyzed using the CONTIN algorithm [93].

The average molecular weight, *M*_W_, and second osmotic virial coefficient, *A*_2_, for the micelles were obtained from the Debye plots determined by concentration dependence of scattering intensity data. According to the Rayleigh-Gans-Debye theory, the scattered intensity of light from a dilute solution of weakly interacting particles with dimensions that are small compared with the wavelength of the incident light (*i.e.*, diameter < λ/20) may be approximated by [94]:

(14) $$\frac{K(c-cmc)}{\Delta R_{\theta }}=\frac{1}{M_{\text{W}}}+2A_{2}(c-cmc)$$

where, *c* denotes the concentration of surfactant, *c-cmc* is the micellar concentration, *A*_2_ is the second osmotic virial coefficient and *K* is an optical constant given by *K* = 4π^2^*n*_0_^2^(d*n*/d*c*)^2^*N*_A_λ_0_^4^ in which *n*_0_ is the refractive index of the solution, *N*_A_ is Avogadro’s number, λ_0_ is the wavelength of the laser, and d*n*/d*c* is the refractive index increment of the micellar solution. The refractive index values of the solvent and micellar solutions were measured using a digital Abbe refractometer (WYA-1S). The refractive index increment was determined by fitting *n* as a linear function of the surfactant concentration. The excess Rayleigh ratio of the micelles (Δ*R*_θ_) is given by:

(15) $$\Delta R_{\theta }=(I-I_{\text{solv}})\frac{R_{\text{tol}}}{I_{\text{tol}}}$$

where *I* is the scattered intensity of the micellar solution, *I*_solv_ is the scattered intensity of the solvent in the absence of micelles, and *I*_tol_ is the scattered intensity of toluene, which was used in our study as a standard. *R*_tol_ is the Rayleigh ratio of toluene (assumed to be 1.3523 × 10^−3^ m^−1^). The scattered light intensity was measured at least four times for each sample. The average error in these repeated measurements was approximately 2%.

The parameter measured directly in the dynamic light scattering experiments (DLS) was the time autocorrelation function of the scattered light. The Brownian motion of micelles in solution is described by the Stokes-Einstein Equation:

(16) $$D_{0}=\frac{k_{\text{B}}\;T}{6\;\pi \;\eta \;R_{0}}$$

where *D*_0_ is the diffusion coefficient, *k*_B_ the Boltzmann constant, *T* the absolute temperature, η the viscosity, and *R*_0_ the equivalent spherical radius of the micelles. Using the data treatment described in previous papers [37,40], fluctuations in scattering intensity were analyzed via the intensity autocorrelation function. The collective diffusion coefficient (*D*_c_) was calculated based on the relation *Γ* = *D*_c_*q*^2^ where *Γ* and *q*^2^ are the decay rate and the square of the amplitude of the scattering wavevector, respectively. The diffusion coefficient thus obtained reflects the diffusion of the micelles as affected by the intermicellar interactions (the so-called collective diffusion). At high micelle concentrations, these interactions are of two types: direct (such as repulsive excluded-volume effects and attractive van der Waals interaction) and hydrodynamic (in which the motions of one particle are communicated to other particles via the flow of the solvent). In the dilute region, the collective diffusion coefficient can be expanded in power series of the surfactant concentration [95], that is,

(17) $$D_{c}=D_{0}[1+k_{\text{D}}\;(c-cmc)]$$

where *k*_D_ is a constant related to the second osmotic virial coefficient (*A*_2_) and the frictional coefficient, which depends on the micelle geometry [72]. *D*_0_ will correspond to the diffusion coefficient in the absence of interaction, that is, at the cmc. The micellar size was measured at least three times for each sample. The average error in these experiments was estimated at 4%.

The dynamic viscosity (η) of the solvent systems was obtained with an automated microviscometer AMVn (Anton Paar: Graz, Austria). The measuring principle of this apparatus is based on Stoke’s law. The apparatus determines the falling time of a small steel ball between a fixed distance into a Peltier-thermostated capillary.

#### 3.2.3. Fluorescence Spectroscopy

Steady-state fluorescence measurements were performed using in a FluoroMax-4 (Horiba Jobin Yvon, Kyoto, Japan) spectrofluorometer in “S” mode, with a 1 cm path-length quartz cuvette. This apparatus is equipped with a Peltier drive that allowed the temperature to be controlled to ±0.01 °C. Fluorescence emission spectra of OTG solutions containing between 1 and 2 μM pyrene were recorded between 360 and 500 nm at an excitation wavelength of 335 nm. From these spectra, the intensities *I*_1_ and *I*_3_ were measured at the wavelengths corresponding to the first and third vibronic bands located near 373 and 384 nm. The ratio *I*_1_/*I*_3_ is the so-called pyrene 1:3 ratio index. The cmc determination for each solvent mixture was repeated at least twice. If an appropriate reproducibility was not obtained, the experiment was repeated a third time.

A diode laser-based luminescence spectrometer (model LifeSpec II, Edinburgh Instruments, Ltd., Livingston, UK) was used to perform all time-resolved fluorescence measurements. A picosecond pulsed diode laser at 405 nm (Edinburgh Instruments, Ltd.) was employed as the excitation source, with emission being recorded at 525 nm. To optimize the signal-to-noise ratio, 10^4^ photon counts were collected in the peak channel. The instrumental response function (IRF) was regularly obtained by measuring the scattering of a Ludox solution. The IRF for this setup was about 230 ps at fwhm. The decay curves were deconvoluted using the FAST software package from Edinburgh Instruments. The intensity decay curves for all lifetime measurements were fitted as a sum of exponential terms:

(18) $$I(t)=\sum_{i}A_{i}\;\text{exp}\left(\frac{t}{\tau_{i}}\right)$$

where *A_i_* is a pre-exponential factor of the component *i* with a lifetime τ*_i_*. In these experiments, the temperature was maintained at the desired value (25 °C) using a Peltier system with an accuracy of ±0.1 °C.

Time-resolved fluorescence anisotropy experiments were carried out by measuring the fluorescence decays for parallel, *I*_VV_(*t*), and perpendicular, *I*_VH_(*t*), polarizations with respect to the vertically polarized excitation beam. The anisotropy decay, *r*(*t*), was obtained using the relation [85],

(19) $$r(t)=\frac{I_{\text{VV}}(t)-G\;I_{\text{VH}}(t)}{I_{\text{VV}}(t)+2G\;I_{\text{VH}}(t)}$$

where the *G* factor was also experimentally determined using a methanolic solution of C153, thus ensuring a very fast rotational relaxation of the probe. A double-exponential decay was required to ensure the best fit of the anisotropy decays in all cases. The statistical criterion for determining the goodness-of-fit of either fluorescence or anisotropy decays was a reduced χ^2^ value of <1.2 and a random distribution of weighted residuals.

## 4. Conclusions

Surface tension, light scattering and fluorescence probe measurements have been carried out on OTG micellar solutions in aqueous solvent media containing varying EG contents. These studies have shown that the addition of EG to the solvent system reduces the solvophobic effect driving the micellization of OTG. As a consequence, the cmc of the surfactant increases and its surface activity decreases. The micellar size, as reported by both hydrodynamic radius and mean aggregation number of micelles, was found to decrease in EG-rich media. In addition, the presence of co-solvent weakens the intermicellar interactions, by reducing of the dielectric constant of the medium. All these effects occurred in the same manner as for the conventional ethoxylated surfactants but to a lesser extent, probably due to the structural differences between their headgroups. Thus, the fact that water is more strongly bound to the sugar headgroups implies that the solvation layer of these micelles is less likely to be altered when the conditions in which micelle formation occurs are modified. The results obtained upon analysis of the rotational dynamics of a hydrophobic probe solubilized in the micellar palisade layer suggest some degree of dehydration in the micellar solvation layer rather than participation of the co-solvent in this region of the micelle.

To the best of our knowledge, this is the first report of the effect of a non-aqueous polar solvent such as EG on the micellization of an alkylglucoside surfactant. This study demonstrates that introduction of a co-solvent modifies the quality of the solvent, thus providing a means to control the self-assembly and other properties of the system. The information presented herein offers new possibilities to modulate the aggregation behavior of OTG for specific applications in a rational manner.

## Figures and Tables

**Table 1:** Effect of EG addition on micellization and adsorption parameters of OTG at 25 °C.

EG (% wt)	(cmc) (mM) a	−Δ*G*_mic_^0^ (kJ/mol)	Γ_max_ 10^3^ (mmol/m^2^)	A_min_ Å^2^/molecule	Π_cmc_ (mN/m)	−Δ*G*_ads_^0^ (kJ/mol)
0	7.9 (8.7)	22.0	3.1	52.9	42.3	35.3
10	9.4 (10.5)	21.4	3.4	49.4	37.7	32.5
20	10.1 (12.6)	21.0	3.6	46.5	33.8	30.5
30	13.2 (16.0)	20.2	3.5	48.0	30.4	28.9
40	18.1 (22.3)	19.2	4.0	41.6	27.2	26.0

a Within parenthesis are the cmc values as obtained by the pyrene 1:3 ratio method.

**Table 2:** Structural parameters of OTG micelles as a function of the EG content at 25 °C. Viscosity of the solvent, η, mean aggregation number, *N*_agg_, hydrodynamic radius, *R*_0_, second osmotic virial coefficient, *A*_2_, coefficient *k*_D_ (see Experimental section), effective radius of micelles, *R*_EFF_, and micellar density, ρ.

EG (% wt)	η (mPas)	*N*_agg_	*R*_0_ (nm)	−A_2_ × 10^9^ (mol m^3^ g^−2^)	−*k*_D_ (L g^−1^)	*R*_EFF_ (nm)	ρ (g cm^−3^)
0	0.89	114	3.5	7.3	0.75	9.6	0.32
10	1.13	99	3.6	4.7	0.27	7.6	0.26
20	1.34	95	2.9	2.6	0.09	6.0	0.48
30	1.82	82	2.7	0.6	0.03	3.4	0.51
40	2.42	45	2.0	0.7	0.02	2.4	0.69

**Table 3:** Anisotropy decay parameters of C153 in micelles of OTG (30 mM) in EG-water mixtures of different composition.

%wt EG	*r*_0_	β	τ_slow_ (ns)	τ_fast_ (ns)	χ^2^	〈τ*_r_*〉 (ns)
0	0.288	0.40 ± 0.01	3.77 ± 0.12	0.66 ± 0.01	1.04	1.90 ± 0.18
10	0.281	0.41 ± 0.01	3.63 ± 0.11	0.61 ± 0.01	1.05	1.90 ± 0.17
20	0.280	0.41 ± 0.01	3.39 ± 0.11	0.62 ± 0.01	1.03	1.76 ± 0.16
30	0.274	0.41 ± 0.01	3.22 ± 0.10	0.59 ± 0.01	1.16	1.66 ± 0.17
40	0.244	0.40 ± 0.02	2.93 ± 0.10	0.52 ± 0.01	1.04	1.45 ± 0.16

**Table 4:** Effect of the EG addition on the microviscosity of OTG micelles obtained from the average reorientation times of C153 in micelles.

%wt EG	〈τ*_r_*〉 (ns)	lang;τ*_r_*〉_P_ (ns)	τ_M_ (ns)	η_m_ (mPa s)
0	1.90	1.90	1800	32.6
10	1.90	1.90	1181	32.6
20	1.76	1.78	131.5	30.5
30	1.66	1.72	50.6	29.5
40	1.45	1.57	19.7	26.9
